# Comparison between Minimally Invasive Plate Osteosynthesis and Conventional Open Plating for Midshaft Clavicle Fractures: A Systematic Review and Meta-Analysis

**DOI:** 10.1155/2019/7081032

**Published:** 2019-10-16

**Authors:** Enzhe Zhao, Rui Zhang, Dou Wu, Yao Guo, Qiang Liu

**Affiliations:** ^1^Shanxi Medical University, Taiyuan, Shanxi 030001, China; ^2^Department of Orthopedics, Shanxi Dayi Hospital Affiliated to Shanxi Medical University, Shanxi Academy of Medical Science, Taiyuan, Shanxi 030032, China

## Abstract

**Objective:**

The aim of this study was to compare the functional outcome and complications in midshaft clavicle fractures receiving minimally invasive plate osteosynthesis and conventional open plating.

**Methods:**

Relevant studies were searched in the databases of Medline, EMBASE, Cochrane Library, Ovid, and Web of Science from inception to March 1, 2019. Pooled data were analyzed with Cochrane Collaboration's Review Manager 5.3.

**Results:**

A total of 7 studies were included, of which 2 were randomized controlled trials, 3 were retrospective cohort studies, and 2 were prospective cohort studies including 316 patients. No statistical differences in functional outcome (weighted mean difference [WMD] = 0.99, *P* = 0.12), operation time (WMD = −10.44, *P* = 0.07) and time to bone union (WMD = −0.23, *P* = 0.70) were observed between the two groups. However, minimally invasive plate osteosynthesis reduced rates of skin numbness (odds ratio (OR) = 0.25, 95% CI : 0.13 to 0.48; *P* < 0.0001) and complications (OR = 0.33, 95% CI : 0.16 to 0.71; *P* = 0.005) compared with conventional open plating.

**Conclusion:**

This systematic review and meta-analysis found no differences in terms of functional outcomes, operation time, and fracture healing time between minimally invasive plate osteosynthesis and conventional open plating. However, minimally invasive plate osteosynthesis had apparent advantages in rates of skin numbness and complications.

## 1. Introduction

The clavicle fractures constitute an estimated 2% to 5% of all fractures in adults [1]. These fractures are most common in younger patients, and are often associated with direct clavicle injuries such as contact sports and motor vehicle accidents. It is estimated that approximately 80% occur in the middle third of the clavicle, of which half are displaced [2]. Traditionally, midshaft clavicle fractures were treated nonoperatively by closed reduction, sling or figure-of-8 harness, and physical therapy, with a low rate of nonunion less than 1% [3]. However, several studies illustrated that the nonunion rate with nonsurgical management is between 15% and 20% [4–6]. Furthermore, a current meta-analysis found that surgical treatment of midshaft clavicle fractures presented a significantly lower nonunion rate compared with nonoperative treatment[7]. Therefore, surgical treatment is often preferred for midshaft clavicle fractures today.

Open reduction and plate fixation is one of most commonly performed surgical techniques for midshaft clavicle fractures. However, conventional open plating (COP) may compromise blood supply, soft tissues, and cause several adverse events especially anterior chest numbness or hypoesthesia [8]. The minimally invasive plate osteosynthesis (MIPO) technique was widely used for lower extremity fractures owing to its role in protecting periosteal blood supply of the fracture area [9]. Recently, MIPO technique has been utilized for the treatment of midshaft clavicle fractures with satisfactory clinical outcomes [10, 11]. To date, several clinical studies indicated that MIPO could achieve similar results with fewer complications compared with COP [12, 13]. However, the optimal surgical approach for midshaft clavicle fractures remains controversial. Based on the information all above, this systematic review and meta-analysis aims to compare functional outcome and complications of MIPO with COP in the treatment of midshaft clavicle fractures.

## 2. Methods

### 2.1. Search Strategy and Article Selection

The literature searches were performed in the following databases: Medline, EMBASE, Cochrane Library, Ovid, and Web of Science from inception to March 1, 2019. The key words used were “clavicle/collarbone/clavicular”, “midshaft/mid-shaft”, “fracture/broken”, “plate”, “open”, and “minimally invasive/MIPO” in combination with the Boolean operators “AND” or “OR”. Search the reference lists of selected articles manually as a secondary source. Articles were not restricted by languages and publication type.

Two reviewers (Enzhe Zhao, Rui Zhang) screened title and abstract of the search results independently, and removed duplicate articles. Both reviewers screened potentially relevant articles in full for evaluation. Disagreements were resolved by discussion with a third reviewer (Dou Wu).

### 2.2. Eligibility Criteria

**Figure 1:**
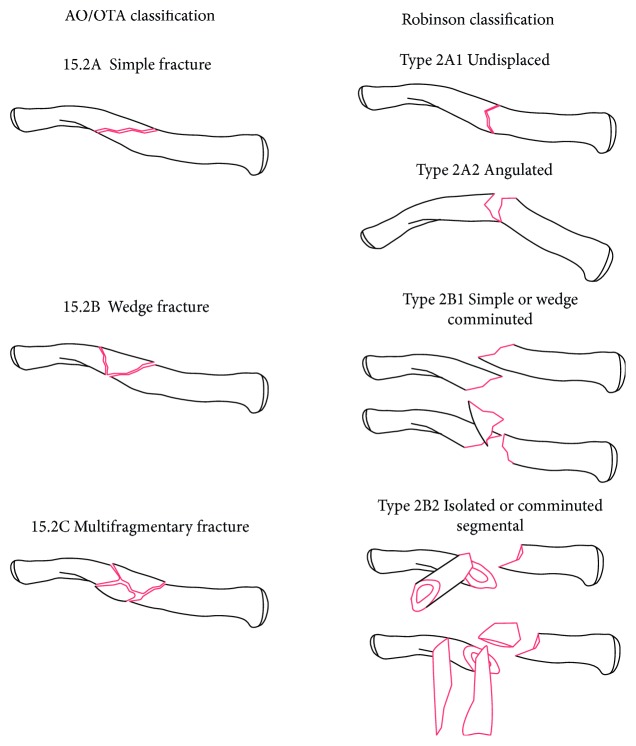
AO/OTA classification and Robinson classification of midshaft clavicle fractures.

The inclusion criteria were as follows: (a) study design: randomized controlled study (RCT) or nonRCT; (b) patients with midshaft clavicle fractures (15.2 according to AO/OTA classification [14] or type 2 according to the Robinson classification [15]) (Figure 1); (c) intervention: MIPO and COP; (d) at least one of following data were reported: functional outcomes, operative time, union time, and complications.

The exclusion criteria were as follows: (a) studies without controlled groups; (b) duplicate publication; (c) pathological fractures; (d) unavailable relevant data.

### 2.3. Quality Assessment

Modified version of the Cochrane Collaboration's tool was applied to assess the risk of bias in RCTs [16]. The Methodological Index for Nonrandomized Studies (MINORS) methodology was used to evaluate other nonRCTs [17]. According to the Cochrane Collaboration recommendations, two reviewers (Enzhe Zhao and Yao Guo) assessed the methodological quality of each included study independently, and a third reviewer (Dou Wu) solved any possible inconsistency.

### 2.4. Data Extraction and Outcome Measurement

A spreadsheet for data extraction was created prior to this study. Two researchers independently extracted the baseline study data as follows: the first author's name, study design, year of publication, interventions, sample size, mean age, follow-up time, operative time, complications, time to bone union, and functional outcomes.

The primary outcome of this meta-analysis was Constant-Murley Shoulder score. Secondary outcomes were operative time, time to bone union, skin numbness/hypoesthesia, and the other complications (e.g., infection, hypertrophic scaring, nonunion, re-fracture, implant failure, skin irritation, and painful shoulder). Fracture union was assessed using radiographic evidence, such as callus formation and bony bridging across the fracture site. Nonunion defined as a lack of complete osseous bridging after 6 months.

### 2.5. Statistical Analysis

The Review Manager software (RevMan 5.3, The Nordic Cochrane Center, The Cochrane Collaboration, Copenhagen, Denmark) was used for data analysis [18]. The weighted mean differences (WMDs) and odds ratios (ORs) were used to represent continuous and dichotomous outcomes, respectively. Data were pooled using the inverse-variance method for continuous outcomes and Mantel-Haenszel method for dichotomous outcomes. All data were reported with WMD or OR and the 95% confidence intervals (CI). Heterogeneity between studies was assessed using Chi-square test and *I*-squared test (*I*^2^). Fixed-effects model was used if no significant heterogeneity existed (*P* > 0.1, *I*^2^ < 50%). If significant heterogeneity was present (*P* < 0.1 or *I*^2^ > 50%), data were rechecked first, then a random-effects model was used when heterogeneity persisted. Sensitivity analysis was evaluated by sequentially removing outlier studies, one at a time.

## 3. Results

### 3.1. Search Results

**Figure 2:**
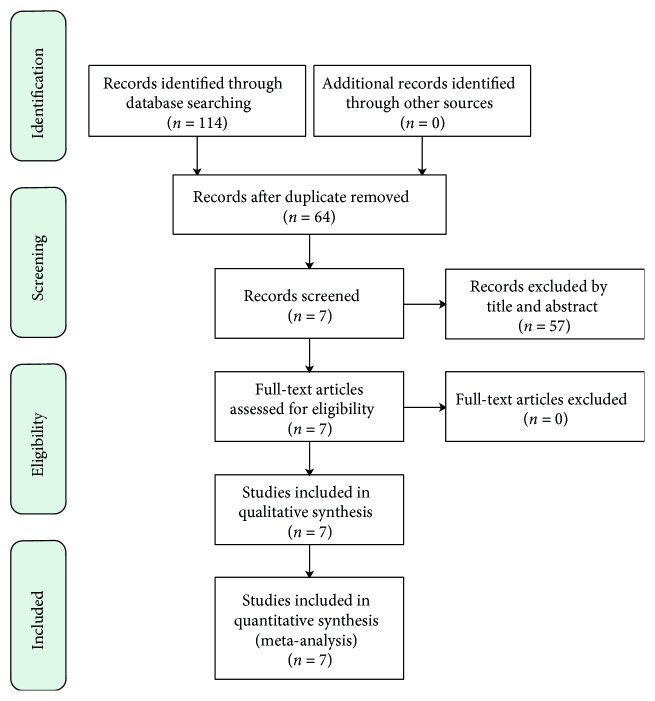
Preferred Reporting Items for Systematic Reviews and Meta-Analyses flow diagram of study selection process.

A systematic search strategy was created and a total of 114 relevant articles were identified. After removal of duplicates, 64 articles were screened based on title and abstract for eligibility, and 7 articles were selected. After reading the full text of these 7 articles, no article was excluded based on the selection criteria. Therefore, 7 articles were included in the systematic review [12,13,19–23] (Figure 2).

### 3.2. Quality Assessment

**Figure 3:**
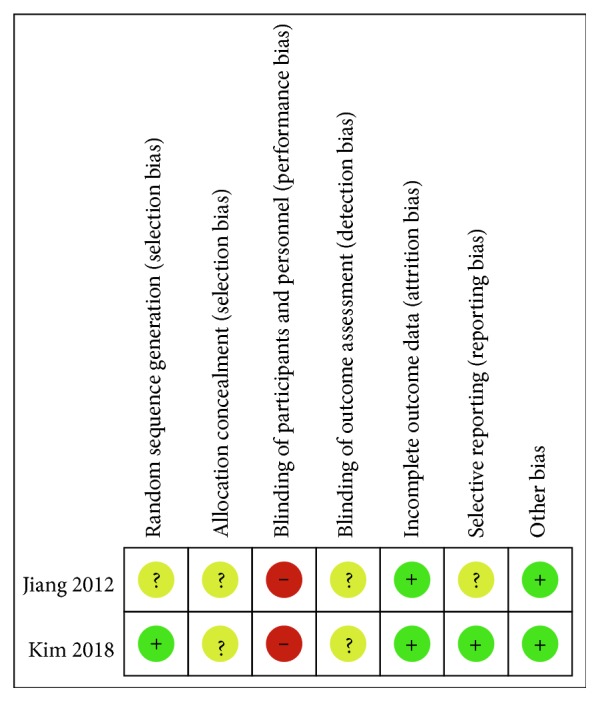
Risk of bias assessment summary of randomized controlled trials. “+” = risk of bias not present, “−” = risk of bias present, and “?” = insufficient information to judge risk of bias.

The quality of RCTs was evaluated by modified version of the Cochrane Collaboration Risk of Bias Tool (Figure 3), and MINORS methodology was used to evaluate nonRCTs (Table 1).

### 3.3. Study Characteristics

A total of 316 patients with midshaft clavicle fractures were involved, including 158 patients treated by COP and 158 patients treated by MIPO. Of the 7 included studies, 2 were randomized controlled trials, 3 were retrospective cohort studies, and 2 were prospective cohort studies. The full characteristics of the included studies are listed in Table 2.

### 3.4. Outcomes of Meta-Analysis

#### 3.4.1. Functional Outcome

**Figure 4:**
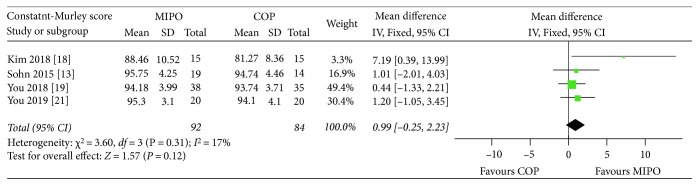
Forest plot diagram of Constant-Murley scores compared between MIPO and COP.

Five studies [12, 13, 20, 21, 23] reported Constant-Murley scores at one-year follow-up. However, one study [12] was excluded from this analysis, for reason that both standard deviation and standard error were not available from the full text. A fixed-effects model was used without heterogeneity (*P* = 0.31,*I*^2^ = 17%). Finally, it was found that the Constant-Murley scores at one-year follow-up did not differ between two groups (WMD = 0.99, 95% CI : −0.25 to 2.23; *P* = 0.12) (Figure 4).

#### 3.4.2. Operation Time

**Figure 5:**
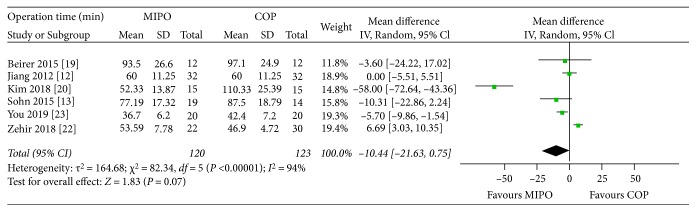
Forest plot diagram of operation time (min) compared between MIPO and COP.

Six studies [12, 13, 19, 20, 22, 23] which involved 243 cases provided data of operation time. The random-effects model was performed due to a remarkable heterogeneity across studies (*P* < 0.00001, *I*^2^ = 94%). No significant difference was found between MIPO and COP in the pooled estimate of operation time (WMD = −10.44, 95% CI : −21.63 to 0.75; *P* = 0.07) (Figure 5). Sensitive analysis showed that the total pooled effect size was greatly affected by the study of Zehir [22] (*I*^2^ = 93%; WMD = −14.77, 95% CI : −28.63 to −0.91; *P* = 0.04).

#### 3.4.3. Time to Bone Union

**Figure 6:**
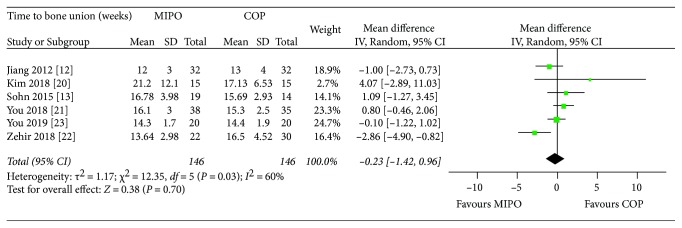
Forest plot diagram of time to bone union (weeks) compared between MIPO and COP.

Time to bone union was reported in all seven studies. However, one study [19] only reported the maximum and minimum values of healing time without mean values and standard deviation, thus a total of six studies [12, 13, 20–23] were included in this analysis. A random-effects model was applied due to the remarkable heterogeneity across studies (*P* = 0.03,*I*^2^ = 60%). There was no significant statistical difference between MIPO and COP regarding time to bone union (WMD = −0.23, 95% CI : −1.42 to 0.96; *P* = 0.70) (Figure 6). In addition, a sensitive analysis by excluding outlier study [22] showed that the result was robust (*I*^2^ = 15%; WMD = 0.20, 95% CI : −0.62 to 1.02; *P* = 0.63).

#### 3.4.4. Skin Numbness

**Figure 7:**
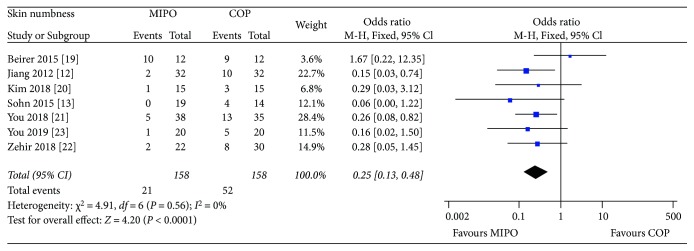
Forest plot diagram of skin numbness compared between MIPO and COP.

Skin numbness or hypoesthesia was assessed in all seven studies with a minimum of 6 months follow-up. A fixed-effects model was performed without heterogeneity (*P* = 0.56,*I*^2^ = 0%). Overall, the pooled results showed that skin numbness occurred more often after COP (OR = 0.25, 95% CI : 0.13 to 0.48; *P* < 0.0001) (Figure 7).

#### 3.4.5. Complications

**Figure 8:**
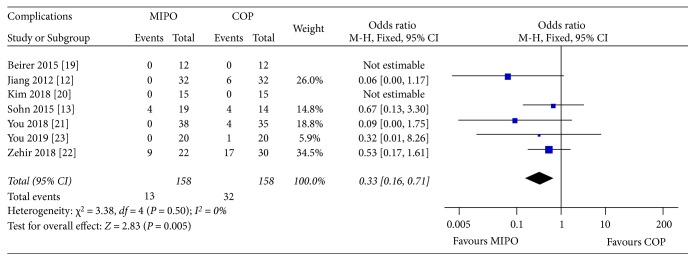
Forest plot diagram of complications compared between MIPO and COP.

The reported complications used for this analysis included infection, hypertrophic scaring, nonunion, re-fracture, implant failure, skin irritation, and painful shoulder except skin numbness/hypoesthesia. A full list of complications can be viewed in Table 3. Two studies [19, 20] reported no complication except skin numbness/hypoesthesia. A fixed-effects model was applied without heterogeneity (*P* = 0.50,*I*^2^ = 0.%). The pooled results showed that complications significantly favored COP (OR = 0.33, 95% CI : 0.16 to 0.71; *P* = 0.005) (Figure 8).

## 4. Discussion

The present systematic review and meta-analysis, comparing MIPO versus COP for the treatment of midshaft clavicle fractures, found no differences in terms of long term functional outcomes, operation time, and time to bone union between MIPO and COP. However, MIPO had apparent advantages in rates of skin numbness and complications. These results suggested that MIPO is a safe surgical treatment of midshaft clavicle fractures with fewer complications.

Postoperative functional recovery, one of the most crucial outcomes, is closely related to the quality of life of patients. Clinical scores such as American Shoulder and Elbow Surgeons (ASES) score, Constant-Murley score and Disabilities of the Arm, Shoulder, and Hand (DASH) were often used to assess the postoperative functional recovery. We also employed the Constant-Murley score, including pain, range of motion and activities of daily living, to evaluate postoperative functional recovery. Although several studies [10, 11, 24–26] have reported the good clinical outcomes of MIPO, this meta-analysis found no difference in terms of long term functional outcomes at one-year follow-up between MIPO and COP (*P* = 0.12).

Two studies [20, 23] showed mean operation time of MIPO was shorter than COP. However, Zehir et al. [22] reported the operation time of COP was shorter. Our meta-analysis found no significant difference in operation time between MIPO and COP (*P* = 0.07). There was a significant heterogeneity between the two groups (*P* < 0.00001, *I*^2^ = 94%), and sensitive analysis showed the total pooled effect size was greatly affected by the study of Zehir [22]. Operation time of COP was shorter than MIPO in the study of Zehir [22] might be due to repetitive fluoroscopy use for fracture reduction and placement of plate and high loss to follow-up (10/32) in the MIPO group.

MIPO technique, used to stabilize acute fractures without extensive soft tissue dissection, was believed to promote bone healing by preserving the enveloped soft tissue and periosteal circulation [10]. However, this meta-analysis found no significant difference in time to bone union between MIPO and COP groups (*P* = 0.70). This might be due to low sample size of included studies and the careful dissection during COP to avoid damaging blood supply. Although there was a significant heterogeneity between the two groups (*P* = 0.03, *I*^2^ = 60%), these findings were reliable because the result of sensitive analysis did not alter significance by excluding the outlier study [22] (*I*^2^ = 15%; WMD = 0.20, 95% CI : −0.62 to 1.02; *P* = 0.63).

Previous studies have shown that anterior chest wall numbness is one of the most common complications in the treatment of clavicle fracture with plate, by reason of damage to branches of supraclavicular nerve [27, 28]. The incidence of skin numbness after COP has been reported to be 12%–83% [28, 29]. Several investigations reported MIPO technique significantly reduced the anterior chest wall numbness compared with COP [19, 21]. Similarly, our meta-analysis found that MIPO was superior to OCP in skin numbness (*P* < 0.0001). Anatomically, no branch of supraclavicular nerve was found within 2.7 cm of the sternoclavicular joint or within 1.9 cm of the acromioclavicular joint [30]. The medial and lateral incision selected by the MIPO technique was precisely within these two areas with no neural branches. Although an additional central incision on the fracture site is needed for anatomical reduction in MIPO (the technique used in studies of Jiang [12], Beirer [19] and Zehir [22]), the incision length and soft tissue forcible retraction in MIPO were still less than COP. These might explain the reason why skin numbness occurred more often in the COP group.

In this meta-analysis, complications such as infection, hypertrophic scaring, nonunion, refracture, implant failure, skin irritation, and painful shoulder were evaluated also. The reason why these complications were pooled together was that the sample size would be smaller if each complication was assessed separately. Pooled results indicated that patients receiving COP had more complications than MIPO, and this was found to be significant (*P* = 0.005). Five of the included studies reported no major complications such as nonunion, re-fracture, and implant failure in both groups [12, 19–21, 23].This might be due to the low sample size of included studies.

To our knowledge, this is the first systematic review and meta-analysis comparing MIPO and COP for the treatment of midshaft clavicle fractures. However, there were several limitations that should be noted. First, there were only two RCTs that met the eligibility criteria, and the sample sizes were small in most studies. Second, the follow-up duration was relatively short and might underestimate the complications. Third, due to the lack of relevant data, we could not perform subgroup analysis according to fracture classification. Finally, the existence of bias, owing to different surgeons and surgical technologies, might have been inevitable in our research.

## 5. Conclusion

This systematic review and meta-analysis found no differences in terms of functional outcomes, operation time, and fracture healing time between MIPO and COP. However, skin numbness and complications appear to occur more frequently when COP is used. High quality clinical trials which include larger sample sizes and longer follow-up time are required to confirm our conclusion.

## Figures and Tables

**Table 1:** Quality assessment of nonrandomized studies (methodological index for nonrandomized studies).

	Beirer 2015 [19]	Sohn 2015 [13]	You 2018 [21]	Zehir 2018 [22]	You 2019 [23]
A clearly stated aim	2	2	2	2	2
Inclusion of consecutive patients	2	2	2	2	2
Prospective data collection	2	0	2	0	0
End points appropriate to the aim of the study	1	2	2	2	2
Unbiased assessment of the study end point	0	0	0	0	0
A follow-up period appropriate to the aims of study	1	2	2	2	2
Less than 5% loss to follow-up	2	2	2	0	2
Prospective calculation of the sample size	0	0	0	0	0
An adequate control group	2	2	2	2	2
Contemporary groups	2	2	2	2	2
Baseline equivalence of groups	2	2	2	2	2
Adequate statistical analyses	2	2	2	2	2
Total score	18	18	20	16	18

The items are scored 0 (not reported), 1 (reported but inadequate), or 2 (reported and adequate).

**Table 2:** Characteristics of the studies.

First author	Year	Study design	Group	Total number	Mean age (years)	Gender (M/F)	Follow-up (months)	Fracture classification	Operative technique
Jiang [12]	2012	RCT	MIPO	32	40	20/12	15	Robinson 2B1, 2B2	A central incision over the fracture site (3 cm), a distal incision (1 cm) and a proximal incision (1 cm)
			COP	32	45	20/12	15		A transverse incision over the fracture site (8–10 cm)
Sohn [13]	2015	RC	MIPO	19	46.79	18/1	17.6	AO/OTA 15.2A, 15.2B, 15.2C	Two small skin incisions (the medial and lateral sides of the clavicle)
			COP	14	44.14	12/2	17.6		A transverse incision over the fracture site
Beirer [19]	2015	PC	MIPO	12	34.92	11/1	6	AO/OTA 15.2A, 15.2B, 15.2C	A central incision over the fracture site, a medial stab incision and a lateral stab incision
			COP	12	41.42	11/1	6		A transverse incision over the fracture site
Kim [20]	2018	RCT	MIPO	15	38.13	10/5	13.33	AO/OTA 15.2A, 15.2B, 15.2C	Two small skin incisions (the medial and lateral sides of the clavicle)
			COP	15	38.15	11/4	13.73		A transverse incision over the fracture site
You [21]	2018	PC	MIPO	38	38.3	20/18	12	Robinson 2A2, 2B1, 2B2	Two small skin incisions (the medial and lateral sides of the clavicle)
			COP	35	36.9	18/17	12		A transverse incision over the fracture site
Zehir [22]	2018	RC	MIPO	22	32.32	12/10	14.56	AO/OTA 15.2B, 15.2C	A central incision over the fracture site, a distal incision and a proximal incision (2–3 cm)
			COP	30	34.7	18/12	14.79		A transverse incision over the fracture site
You [23]	2019	RC	MIPO	20	37.2	11/9	12	Robinson 2A2, 2B1, 2B2	Two small skin incisions (the medial and lateral sides of the clavicle)
			COP	20	35.1	13/7	12		A transverse incision over the fracture site

M = males, F = females, RCT = randomized controlled trial, RC = retrospective cohort, PC = prospective cohort, MIPO = minimally invasive plate osteosynthesis, COP = conventional open plating, OTA = orthopaedic Trauma Association.

**Table 3:** Reported complications between MIPO and COP groups.

Study	MIPO	COP
Jiang 2012 [12]	—	5 Hypertrophic scarring
		1 Painful shoulder
Sohn 2015 [13]	1 Implant failure or screw loosening^a^	1 Implant failure or screw loosening^b^
	2 Skin irritation or discomfort due to plate prominence	3 Skin irritation or discomfort due to plate prominence
	1 Nonunion	
Beirer 2015 [19]	—	—
Kim 2018 [20]	—	—
You 2018 [21]	—	4 Hypertrophic scarring
Zehir 2018 [22]	1 Infection	2 Infection
	3 Skin irritation	4 Skin irritation
	4 Painful shoulder	7 Painful shoulder
	1 Implant failure^a^	2 Implant failure^a^
		2 Nonunion
You 2019 [23]	—	1 Infection
Total	13	32

^a^Need operation, ^b^not need operation.
